# Telerehabilitation Feasibility in Total Joint Replacement

**DOI:** 10.5195/ijt.2017.6235

**Published:** 2017-11-20

**Authors:** MARK J. NELSON, KAY M. CROSSLEY, MICHAEL G. BOURKE, TREVOR G. RUSSELL

**Affiliations:** 1 PHYSIOTHERAPY DEPARTMENT, QEII JUBILEE HOSPITAL, METRO SOUTH HEALTH, BRISBANE, AUSTRALIA.; 2 FACULTY OF HEALTH AND BEHAVIOURAL SCIENCES, UNIVERSITY OF QUEENSLAND, BRISBANE, AUSTRALIA.; 3 SCHOOL OF ALLIED HEALTH, COLLEGE OF SCIENCE, HEALTH AND ENGINEERING, LA TROBE UNIVERSITY, BUNDOORA, VICTORIA, AUSTRALIA

**Keywords:** Hip replacement, Knee replacement, Telerehabilitation, Telemedicine, Total joint replacement

## Abstract

Despite documented benefits, many Total Joint Replacement (TJR) patients find it difficult to access rehabilitation following discharge from hospital. One solution to improve access for TJR patients is telerehabilitation. This study aimed to assess the feasibility of introducing a telerehabilitation program for TJR patients. TJR patients at QEII Jubilee Hospital were invited to complete a questionnaire regarding their access, feelings towards and preferences in using technology. Seventy-five patients were recruited. Most patients had computer access (72%) and internet (69%) at home. Sixty-five percent of participants were willing to participate in telerehabilitation. A significant difference was found between older and younger patients. Watching videos on an electronic device was the preferred method for a technology-based home exercise program and phone call the preferred method of communication. Results indicate telerehabilitation in the TJR population is feasible from the perspective of access to, feelings toward, and preferences for technology.

Total joint replacement (TJR) is the gold standard for management of severe hip and knee osteoarthritis (OA). In the last decade there has been a 50% rise in the number of total hip replacements (THR) and total knee replacements (TKR) undertaken in Australia with over 90,000 THRs and TKRs performed in in 2014 (Australian Orthopaedic Association National Joint Replacement Registry - Annual Report 2015, 2015). Physical rehabilitation programs for TJR patients following discharge from hospital have been shown to improve walking distance (Bruun-Olsen, Heiberg, Wahl, & Mengshoel; Jakobsen, Kehlet, Husted, Petersen, & Bandholm, 2014; Maire et al., 2004; Wang, Gilbey, & Ackland, 2002), gait speed (Unlu, Eksioglu, Aydog, Aydog, & Atay, 2007; Wang et al., 2002) and muscle strength (Suetta et al., 2004; Unlu et al., 2007). Many TJR patients find it difficult to access health care once discharged from hospital. The elderly demographic coupled with the risk of THR dislocation post-operatively can make driving and transportation difficult (Stergiou-Kita & Grigorovich, 2014). Access to rehabilitation programs is compounded by the financial cost to the patient (Dansky, Palmer, Shea, & Bowles, 2001) and health systems to provide domiciliary services in conjunction with or in lieu of centre based care. For patients living outside metropolitan areas, access issues become magnified due to travelling distances and time for either the patient or treating clinician (“Australian Institute of Health and Welfare. Physiotherapy Labour Force 1998. Canberra: Australian Institute of Health and Welfare,” 2000). The geographical remoteness of regional and rural areas, and a relative shortage of physiotherapists compared to metropolitan areas pose additional barriers for patients to access rehabilitation programs (Theodoros, Russell, & Latifi, 2008).

One solution to improve access for TJR patients is telerehabilitation. Telerehabilitation is defined by Rosen (1999) as the delivery of medical rehabilitation services at a distance using electronic information and communication technologies. There is a growing body of literature supporting the use of telerehabilitation in a diverse range of clinical scenarios. Rehabilitation for neurological conditions, cardiac conditions, musculoskeletal conditions, spinal cord injuries, speech-language impairments and orthopaedic conditions have all been investigated with comparable results to traditional rehabilitation (Cottrell, Galea, O’Leary, Hill, & Russell, 2016; Dorstyn, Mathias, & Denson, 2013; Hwang, Bruning, Morris, Mandrusiak, & Russell, 2015; Jiang, Xiang, Gao, Guo, & Liu, 2016; Regina Molini-Avejonas, Rondon-Melo, de La Higuera Amato, & Samelli, 2015; Steins, Dawes, Esser, & Collett, 2014). The majority of research in post-operative orthopaedic telerehabilitation has focused on the total knee replacement (TKR) population. Multiple randomised controlled trials (RCTs) have compared telerehabilitation programs to conventional programs for post-operative rehabilitation in TKR patients (Piqueras et al., 2013; Russell, Buttrum, Wootton, & Jull, 2011; Tousignant, Moffet, et al., 2011). Russell et al. demonstrated TKR patients undertaking a six week telerehabilitation program obtained comparable outcomes to those receiving traditional in-person rehabilitation with respect to flexion and extension range of motion, muscle strength, limb girth, pain, mobility and quality of life (Russell et al., 2011). Tousignant et al. reinforced these findings concluding that home telerehabilitation is as effective as usual care in reducing disability (range of motion, balance and muscle strength) and improving function (knee function, walking and autonomy) after two months of treatment (Tousignant, Moffet, et al., 2011). This was further strengthened by Piqueras et al. who demonstrated a two week interactive telerehabilitation program is at least as effective as conventional therapy (Piqueras et al., 2013). In addition to achieving comparable outcomes to conventional rehabilitation, patients participating in telerehabilitation reported high levels of satisfaction with their program (Russell, Buttrum, Wootton, & Jull, 2004; Tousignant, Boissy, et al., 2011). Systematic reviews and meta-analyses by Jiang (Jiang et al., 2016) and Shukla (Shukla, Nair, & Thakker, 2017) both recommended telerehabilitation for TKR patients as a practical alternative to conventional face-to-face rehabilitation in TKR.

There are various outcomes that determine the success of telerehabilitation programs: clinical outcomes related to physical, functional, and psychological outcomes; cost analysis from the perspective of both the provider and user; and process measures related to service delivery such as uptake, attendance, satisfaction and adherence to programs. While research reports high levels of satisfaction with telerehabilitation programs, (Lemaire, Boudrias, & Greene, 2001; Mashima et al., 2003; Vesmarovich, Walker, Hauber, Temkin, & Burns, 1999) the successful implementation of a telerehabilitation program is often dependent on initial consumer uptake. This can prove challenging as access to appropriate technology can be difficult, particularly in the elderly population.

The aim of this study was to assess the feasibility of introducing a telerehabilitation program for post-operative TJR patients. This was done via a survey of the TJR population exploring consumer access, feelings toward, and preferences in using technology.

## METHODS

### STUDY DESIGN

Ethical approval to conduct this study was obtained from the Metro South Human Research Ethics Committee (HREC No. HREC/13/QPAH/235) and University of Queensland Medical Research Ethics Committee.

A self-administered questionnaire was developed using frameworks outlined by Frazer (Frazer, 2000) and Oppenheim (Oppenheim, 1992). It consisted of 12 closed questions pertaining to patients’ access, feelings toward, and preferences in using technology (Appendix 1). Questions relating to patients’ feelings towards technology were rated using a 5 point Likert scale ranging from ‘strongly agree’ (5) to ‘strongly disagree’ (1). Questions relating to technology use as part of a telerehabilitation program were ranked in order of preference with 1 being the most preferred option. Four options were provided for communication preferences and 3 for technology-based home exercise program (HEP) preferences. The questionnaire was subject to a pilot process where it underwent review by four TJR patients for readability, layout, language and clarity of questions. Suggestions from the pilot process were incorporated into the final questionnaire.

### PARTICIPANTS

Participants were recruited from the QEII Jubilee Hospital, Brisbane, Australia from December 2014 to February 2016. To be eligible for inclusion, patients must have undergone an elective THR or TKR and been able to provide informed consent. Patients receiving emergency THR or TKR following trauma were not included. Eligible patients were approached by their treating physiotherapist to obtain written consent for participation in the study. Consenting patients were provided a paper questionnaire which they completed independently during their post-operative in-patient stay.

### DATA ANALYSIS

Quantitative data from closed ended questions, Likert scales and preference rankings were analyzed using descriptive statistics which consisted of frequency distribution, percentages and graphical representation of the data. Older and younger age groups, and having access to a computer or not were explored for differences using McNemar and Mann Whitney U tests. McNemar tests were performed for analysis of access to technology where responses were limited to yes or no. Mann Whitney U tests were used for Likert scales and preference rankings. Data was analyzed in two age groups, 65 years and younger, and older than 65 years. Sixty-five was selected as the age cut off based on criteria used by the Australian Government for determining senior citizen status.

## RESULTS

**Figure 1:**
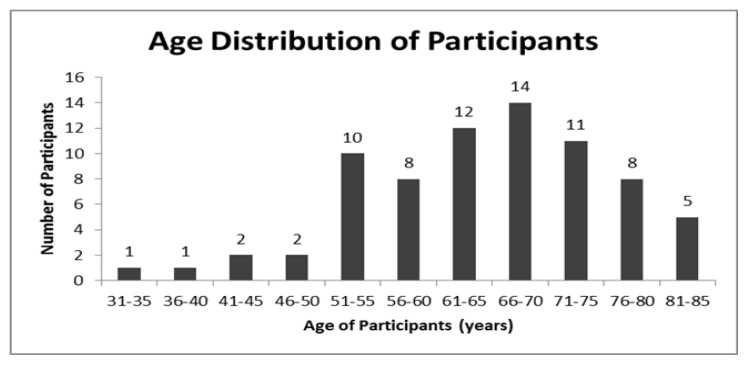
Age distribution of participants.

Seventy-eight patients were approached for consent to participate in the study. Seventy-five (96%) were recruited (32 male, 43 female) with a mean age of 65.0 (35–85) (Figure 1). Thirty-seven (49%) of participants fell in the 65 years and younger age group with a mean age of 56.4 years old. Thirty-eight (51%) participants were aged 66 years or older with a mean age of 73.3 years old.

Fifty-nine (79%) participants completed all questions on the questionnaire. Where a participant had not fully completed a section, their data was omitted from analysis of that section only. Sections 1, 2 and 4 were all fully completed. Sixteen participants did not fully complete section three. Section three required participants to rank, in order of preference, four different communication options and three different home exercise program options that utilised technology to facilitate their rehabilitation. Of the sixteen participants who did not answer these questions completely, twelve were aged 66 years or above and only four owned any type of technology (e.g., computer, tablet, smart phone).

### ACCESS

**Figure 2:**
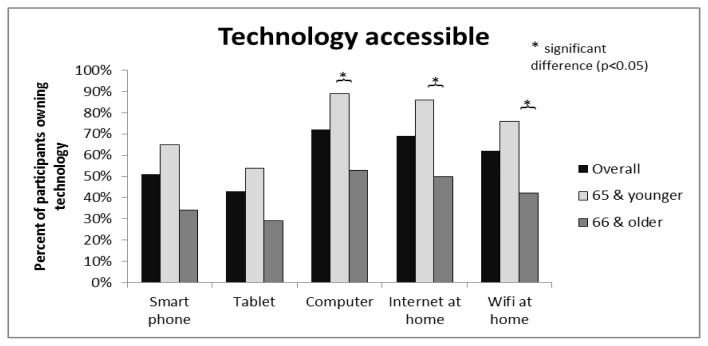
Access to technology in own home of TJR population.

Computers were the most common form of technology accessible in participants’ homes with 72% reporting access. Ninety-five percent of these were connected to the internet. Smart phones (51%) and tablets (43%) were less accessible to participants. Sixty-nine percent of all participants had internet access at home and 62% had wi-fi internet. A significant difference was found between age groups 65 years and younger and 66 years and older for access to a computer, internet and wi-fi at home (Figure 2).

### FEELINGS TOWARD TECHNOLOGY

The majority of participants were both willing to participate (65%) and reported they would feel safe participating (65.8%) in a telerehabilitation program as described in the questionnaire (Appendix 1). Forty-three percent reported feeling apprehensive towards technology and approximately 40% reported avoiding technology. Only 35% reported feeling confident using technology (Table 1)

### PREFERENCES

Overall, watching videos on a device was heavily favoured as the preferred method of a technology facilitated HEP with 71.4% selecting this as t the preferred method for communication with their physiotherapist.

Preferences for using technology were also analyzed by age group and computer access at home. The only finding of statistical significance for participants were more likely to select a phone call as their preferred method of communication compared to the younger cohort (Table 2).

## DISCUSSION

The demographics of participants in this study are reflective of the TJR population reported nationally.

Approximately 70% of participants had access to a computer connected to the internet in their own home, 51% to a smart phone and 43% to a tablet device. A computer connected to the internet would commonly be considered the minimum requirement to participate in a telerehabilitation program. The high levels of access to a computer with internet suggests that purely from an access point of view, telerehabilitation would be feasible in the TJR population. This is particularly true for the younger TJR population of which approximately 85% reported access to an internet connected computer. For the 30% who did not own an internet connected computer, telerehabilitation remains a potential option, however appropriate equipment would need to be supplied by health care providers. Equipment provision brings with it multiple aspects for consideration. Costs associated with purchasing, maintaining and insuring hardware, as well as logistics regarding provision, monitoring, and return of equipment should all be considered as these may impact on the feasibility of implementing a telerehabilitation service.

Having access to a computer at home and participant age were both strongly associated with participants’ feelings towards technology and telerehabilitation. Access to a computer at home appeared to be the strongest predictor. There was a significant difference between those with and without a computer at home in all questions pertaining to participants’ feelings towards technology. Participants with access to a computer were more interested in learning and more confident with technology, avoided technology less, and were more likely to participate and feel safe undertaking a telerehabilitation program. When considering age groups, participants older than 65 years were less likely to participate in telerehabilitation than those under 66 years, however no significant difference was found with respect to their feelings of safety undertaking telerehabilitation. Their unwillingness to participate is more likely linked to the fact they are significantly more apprehensive and less confident with technology than their younger counterparts. This shouldn’t necessarily exclude this age group from being considered for telerehabilitation programs. Despite being less willing to participate, participants from the older age group were as likely to want to learn more about technology as the younger group.

The findings mentioned above are somewhat predictable. It is natural for participants with access to technology in their own homes to be more comfortable with its use, and therefore more willing to accept technology based rehabilitation. Similarly, older participants have likely had less exposure to technology, which may contribute to their feelings of apprehension, lack of confidence, and unwillingness to participate in telerehabilitation. The fact that no significant difference was found in wanting to learn more about technology between age groups is worth noting. Evidence demonstrates that computer training can significantly reduce computer anxiety and significantly increase computer interest and efficacy in older adults (Xie & Bugg, 2009). This implies that providing patients with training and education about relevant technology may increase uptake of telerehabilitation programs. Despite only 35% of all participants reporting they felt confident using technology, 65% of all participants were willing to participate in a telerehabilitation program. Sixty percent of participants reported they were keen to learn more about technology suggesting this may be a better predictor of willingness to participate. Currently, 65% of TJR patients would be willing to participate in a telerehabilitation program, and considering the heightened access to technology of future generations, this figure will only increase with time.

Regardless of age or access to technology in the home, participants preferred a phone call as the favoured method of communication with a physiotherapist. It is reasonable to assume all participants own a phone, and therefore ranked this option highly compared to more unfamiliar forms of communication.

With the wide range of technologies currently available to healthcare providers, participant preferences should not impact the ability to implement telerehabilitation programs, and rather should help guide healthcare providers in tailoring programs towards consumers’ preferences. Despite the majority of participants preferring communication via phone-call, physiotherapists should give consideration to the need for visual interaction and feedback when considering implementing telerehabilitation programs.

The results of our study can be used to consider the practicalities of implementing previously published telerehabilitation interventions. Key elements from existing telerehabilitation RCTs (Piqueras et al., 2013; Russell et al., 2011; Tousignant, Moffet, et al., 2011) include the provision of equipment and the ability for the intervention to be delivered in the patient’s home. All three RCTs investigating telerehabilitation in the TKR population provided patients with the required equipment to participate. While commonplace for research purposes, equipment provision could be considered a genuine barrier by healthcare providers looking to implement telerehabilitation models. Access to appropriate equipment is also closely linked with the ability to deliver telerehabilitation directly into patients’ homes, an important aspect of telerehabilitation. Telerehabilitation interventions not delivered directly into patients’ homes are less attractive to patients. Of existing trials, only Tousignant (Tousignant, Moffet, et al., 2011) delivered all telerehabilitation interventions into the participants’ homes. Piqueras (Piqueras et al., 2013) delivered the first five interventions on-site followed by five directly into the participants’ homes, whereas Russell (Russell et al., 2011) delivered all interventions on-site in an isolated, simulated home environment. This study found that approximately 70% of TJR patients have access to an internet connected computer in their home. This suggests that equipment provision by healthcare providers may not be required for the successful implementation of telerehabilitation models and that it is realistic that these models could be delivered directly into the homes of the TJR population.

Additionally, our results could help inform the planning and configuration of future telerehabilitation services. Approximately 65% of elective THR and TKR patients would be willing to participate in a telerehabilitation program following discharge from the hospital. In addition, almost all of these patients would feel safe receiving their rehabilitation this way. This level of patient uptake demonstrates that health care providers can consider telerehabilitation as a genuine healthcare delivery option in the TJR population.

The results of our study indicate telerehabilitation in the TJR population is feasible from the perspective of access to, feelings toward, and preferences for technology. To maximise uptake of programs, healthcare providers looking to implement telerehabilitation should consider patients’ age, access to technology at home, training requirements, and preferences for technology. Healthcare providers should consider the need to: provide equipment (especially to patients aged 66 years and older); train patients in use of technology to increase familiarity and uptake; utilize phone communication where possible, considering the needs of both healthcare provider and patient; and employ exercise-based programs that utilize videos that patients can view at home.

## Figures and Tables

**Table 1:** Feelings towards Telerehabilitation and Technology

Question	All Participants	Participants by Age					Participants by Computer Access				
		65 & younger		66 & older		P Value	Computer at home		No computer at home		P Value
	Agree & strongly agree	Agree & strongly agree	Mean response on 5 point Likert scale	Agree & strongly agree	Mean response on 5 point Likert scale		Agree & strongly agree	Mean response on 5 point Likert scale	Agree & strongly agree	Mean response on 5 point Likert scale	
Willing to participate in telerehabilitation	64.8%	72.2%	3.8	55.3%	3.1	0.009	83.0%	3.9	19.0%	2.2	0.000
Would feel safe participating in telerehabilitation	65.8%	69.4%	3.8	60.5%	3.3	0.118	79.2%	3.8	30.0%	2.7	0.000
Apprehensive towards technology	42.9%	28.6%	2.5	54.3%	3.4	0.009	34.6%	2.7	70.6%	3.8	0.004
Avoids technology as unfamiliar	39.5%	25.0%	2.3	54.3%	3.3	0.003	32.1%	2.5	64.7%	3.6	0.003
Avoids technology for fear of making mistakes	40.3%	22.2%	2.3	58.3%	3.2	0.004	32.1%	2.5	64.7%	3.5	0.012
Confident using technology	34.8%	50.0%	3.3	19.4%	2.4	0.004	43.4%	3.2	11.1%	1.9	0.000
Interested in learning more about technology	60.0%	69.4%	3.6	50.0%	3.1	0.103	69.2%	3.7	35.3%	2.4	0.000

**Table 2:** Preferences for Using Technology to Facilitate HEP and Communicate with Physiotherapist

Technology Option	All Participants	Participants by Age		Participants by Computer Access							
		65 & younger		66 & older		P Value	Computer at home		No computer at home		P Value
	First preference	First preference	Mean preference ranking (HEP 1–3, Communication 1–4)	First preference	Mean preference ranking (HEP 1–3, Communication 1–4)		First preference	Mean preference ranking (HEP 1–3, Communication 1–4)	First preference	Mean preference ranking (HEP 1–3, Communication 1–4)	
HEP - watching videos on a device	71.4%	80.0%	1.2	65.4%	1.4	0.204	76.0%	1.3	63.6%	1.5	0.379
HEP - receiving HEP via email	15.9%	11.4%	2.2	23.1%	1.9	0.080	18.0%	2.1	18.2%	2.0	0.765
HEP - using gaming console	9.5%	8.6%	2.6	11.5%	2.7	0.387	8.0%	2.7	18.2%	2.5	0.799
Communication - video-call	20.6%	27.8%	2.8	11.5%	3.0	0.887	23.1%	2.9	9.1%	2.9	1.00
Communication - phone-call	57.1%	41.7%	1.9	76.9%	1.3	0.006	51.9%	1.7	81.8%	1.2	0.069
Communication - email	6.3%	8.3%	2.8	3.8%	2.9	0.774	7.7%	2.8	0.0%	3.2	0.149
Communication - text message	15.9%	22.2%	2.4	7.7%	2.8	0.203	17.3%	2.6	9.1%	2.7	0.758
